# Socioeconomic Status Across the Life-Course and Frailty in Older Age: Evidence From Switzerland

**DOI:** 10.3389/ijph.2025.1608102

**Published:** 2025-06-09

**Authors:** Yves Henchoz, Sarah Fustinoni, Laurence Seematter-Bagnoud, Mauricio Avendano

**Affiliations:** ^1^ Department of Epidemiology and Health Systems, Unisanté, University Centre for Primary Care and Public Health, Lausanne, Switzerland; ^2^ Service of Geriatric Medicine and Geriatric Rehabilitation, Lausanne University Hospital, Lausanne, Switzerland; ^3^ Department of Social and Behavioural Sciences, Harvard School of Public Health, Boston, MA, United States

**Keywords:** frailty, socioeconomic status, cohort study, older adults, Switzerland

## Abstract

**Objectives:**

This study examines how different measures of socioeconomic status (SES) across childhood and adulthood relate to frailty in older age.

**Methods:**

Data came from the Lausanne cohort 65+ (Lc65+), a population-based study of approximately 4,500 older adults followed over 20 years. SES measures included education in young adulthood, occupational class in midlife, and specific early old-age factors: perceived income, wealth, financial strain, and receipt of financial subsidies. Frailty trajectories over a 10-year period were assessed using Fried’s frailty phenotype and group-based trajectory modeling. Logistic regression models adjusted for sex, age, cohort, living situation, marital status, and number of children.

**Results:**

Lower education, occupational class, financial strain, and financial subsidies in older age were each independently associated with higher frailty risk at ages 65–70. Financial strain and financial subsidies in early old age increased odds of medium- (aOR, 1.48–1.69) and high-frailty (aOR, 2.07–2.28) trajectories.

**Conclusion:**

SES across the life course strongly correlates with frailty in early old age. Early interventions and financial protection policies in older age could help mitigate frailty risk and SES-related frailty inequalities.

## Introduction

Frailty is a state of progressive decrease in physiological reserves that occurs with ageing, predisposing to increased vulnerability to stressors [1]. It is a strong predictor of adverse ageing outcomes such as disability, cognitive decline, institutionalization, and death [2]. The two main assessment tools are the physical frailty phenotype operationalized by Fried et al., based on five frailty criteria [1], and the frailty index proposed by Rockwood, based on a comprehensive geriatric assessment of individual deficits [3]. Frailty is related to but conceptually distinct from multimorbidity—the co-occurrence of two or more chronic diseases—and sarcopenia, which specifically refers to age-related loss of muscle mass and function [4, 5]. As a broader syndrome that involves multiple physiological domains beyond medical conditions and muscle deterioration, the concept of frailty is highly useful in clinical practice and has significant implications for public policies in the context of population ageing [6].

It is not fully understood why some individuals become frail and experience worse trajectories than others, yet socioeconomic status (SES) often emerges as a strong predictor of frailty outcomes. A recent systematic review concluded that lower education, income and occupational class are all associated with worse frailty outcomes [7]. A limitation of earlier longitudinal studies is their predominant focus on a single pattern of frailty change over time, often assuming a linear increase in frailty with age [8]. This approach fails to account for the diverse range of frailty trajectories in the population. Frailty progression with age is highly heterogeneous and characterized by dynamic changes [9]. While some individuals exhibit a linear increase in frailty, others remain non-frail or experience a sharp increase in frailty. From this perspective, only a few studies have reported socioeconomic inequalities in the likelihood of following unfavorable frailty trajectories in Australia [10], Korea [11], Taiwan [12], and the United States [13, 14].

A second limitation of earlier studies on SES and frailty in older age is the fact that few of them included multiple measures reflecting SES at various life stages. An increasing body of research suggests that frailty in older age may be associated with risk factors during childhood and early adulthood. Among these, socioeconomic circumstances in early life have been proposed as a potential risk factor for frailty in older age, yet the independent association between life-course measures of SES and frailty trajectories still needs systematic examination. This approach could better elucidate how socioeconomic inequalities in frailty develop over the life course and why certain subgroups of the older population become increasingly frail at a faster rate than others.

The aim of this study is to examine how SES measures at different points in the life-course are related to frailty trajectories after the age of 65. We hypothesize that SES measures during childhood are as strong predictors of frailty trajectories as adult SES measures, as SES at different life stages may influence the risk of frailty through different mechanisms.

## Methods

### Study Sample

Data came from the Lausanne cohort 65+ (Lc65+), a longitudinal population-based study of ageing in community-dwelling older adults living in the city of Lausanne, Switzerland. Details of the Lc65+ study are available elsewhere [15, 16]. Briefly, three random samples totaling 4,731 persons aged 65–70 were enrolled in 2004, 2009, and 2014. The following year, they were invited at the study center to complete a frailty phenotype baseline assessment. Follow-up includes annual questionnaires and triennial assessments of the frailty phenotype. The current analyses used 10-year data from the first two cohorts. Supplementary Figure S1 illustrates the selection procedure of study participants. The study protocol and informed consent were approved by the Ethics committee for human research of the canton Vaud (19/04).

### Measures

#### Frailty

Frailty was assessed according to the five criteria of the phenotype described by Fried et al.: shrinking, weakness, exhaustion, slowness and low activity [1, 16]. The frailty phenotype is the most commonly used measure of frailty and has demonstrated high validity and reliability [17, 18]. A frailty score that ranged from 0 to 5 was constructed based on the number of criteria met divided by the number of criteria assessed, multiplied by 5. The frailty score was calculated only if a minimum of three criteria were assessed. Imputations of missing frailty scores were performed according to the following rules applied in the following order of priority: the maximum value of 5 was assigned in case of death; a score of 4 was assigned to participants admitted to a nursing home (until the next non-missing value, end of follow-up, or death), to reflect a state of advanced frailty in institutionalized participants whose phenotype could not be assessed; finally, when the scores of the previous and following assessments were available, the imputed value was the average of the two scores. When used as an adjustment variable (see statistical analysis), the baseline frailty score was dichotomized into “non-frail” (score 0) versus “(pre-)frail” (score >0).

#### Measures of Socioeconomic Status (SES)

All SES measures were assessed at baseline (i.e., 2004–2005 for cohort 1; 2009–2010 for cohort 2). From a life course perspective, we incorporated several measures capturing SES across young adulthood, adulthood, and early old age.

1) Educational attainment, measured by the highest level of education achieved, further classified according to the International Standard Classification of Education (ISCED) as a) basic compulsory (ISCED level 0–2); b) apprenticeship (ISCED level 3); c) post-compulsory schooling (ISCED level 4–8) [19];
2) Longest-held occupation, further categorized into a) manager, self-employed, liberal profession, director; b) skilled worker/employee, farmer; c) non-skilled worker/employee; d) no professional activity;
3) Perceived current relative income as a) clearly higher; b) rather higher; c) rather lower; d) clearly lower compared to same age peers;
4) Perceived current relative wealth as a) clearly higher; b) rather higher; c) rather lower; d) clearly lower compared to same age peers;
5) Current financial strain, measured by replying ‘yes’ to the question ‘Are you sometimes struggling to make ends meet?’;
6) Receipt of financial subsidies, measured by asking participants to report whether they received any type of Government benefits, which are only available to households or individuals classified below a low-income threshold.

#### Other Variables

Demographic variables included sex (males; females), age (years), living alone (no; yes), marital status (single; married; separated or divorced; widowed), and the number of children categorized into 0, 1, 2, or 3+. Subjective health was assessed as very good, good, average, poor, or very poor. The number of chronic conditions ever diagnosed by a physician (hypertension, coronary heart disease, other heart diseases, stroke, diabetes mellitus, chronic respiratory disease, osteoporosis, arthritis, cancer, gastrointestinal ulcer and Parkinson’s disease) was categorized into 0, 1, or 2+. Smoking history was defined as current, former, or never smoking. Problematic alcohol history was assessed as yes or no.

### Statistical Analysis

Baseline characteristics of non-frail and (pre)frail groups were compared using Pearson Chi-squared and Student’s t tests. Associations between socioeconomic characteristics and baseline frailty were assessed using logistic regression models adjusted for sex, age, cohort, living alone, marital status, and number of children. First, separate models were built for each socioeconomic measure. Then a single model included SES measures at all life stages to estimate their mutually adjusted association with baseline frailty. To avoid overadjustment due to multiple subjective SES measures in early old age (i.e., income, wealth, and financial strain), income and wealth were not entered in this mutually adjusted model.

Frailty trajectories were identified using group-based trajectory modelling (GBTM), which we have extensively applied to the Lc65+ data [20]. In brief, GBTM is based on a finite set of polynomial functions of time to model latent groups with similar trajectories. The number and shapes of frailty trajectories was based on the Bayesian information criterion (BIC) and Bayes factors. As dropouts are generally more frequent in individuals with health and socioeconomic vulnerabilities, the missing at random assumption may generate biased estimates of trajectory group size. Therefore, we used a GBTM extension that models the probability of dropout because of death or illness as a function of time and two prior observed outcomes using a logit distribution [21]. Associations between socioeconomic characteristics and frailty trajectories were tested using logistic regression, also using separate models and a mutually adjusted model (see above) and adjusting for the same variables plus baseline frailty.

A sensitivity analysis was carried out to explore the robustness of the results when including individuals lost to follow-up for reasons other than death or illness [22]. A second sensitivity analysis was performed to examine the potential influence of chronic conditions, smoking history, and problematic alcohol history on the main findings.

## Results

Compared to participants included in the main analyses (N = 2,241), excluded participants (N = 812) were slightly older (69.1 years versus 68.9 years, p = 0.003) and rated their health less favorably (p < 0.001), but they did not differ in terms of sex (p = 0.670) and cohort (p = 0.680), as indicated in Supplementary Table S1.

Table 1 provides the baseline prevalence of frailty according to population characteristics. The prevalence of (pre)frailty was higher in females (p = 0.006), older individuals (p < 0.001), participants living alone (p < 0.001), those reporting a marital status other than married (i.e., single, separated, divorced, or widowed, p < 0.001), individuals with multimorbidity (p < 0.001), and those with a history of problematic alcohol consumption (p < 0.001). All SES measures were associated with (pre)frailty at baseline. That is, (pre)frail individuals were more likely to have only basic compulsory education (p = 0.003); to have no professional activity or be non-skilled (p = 0.002); to perceive that their income (p < 0.001) and wealth (p < 0.001) was lower than that of their peers; and to experience financial strain (p < 0.001) and receive financial subsidies (p < 0.001).

**TABLE 1:** Baseline prevalence of frailty according to population characteristics (Lausanne cohort 65+, Switzerland. 2004–2019).

Variables	Non-frail n = 1,600	Pre-frail/Frail n = 641	Total N = 2,241	p-value^a^
Demographic and health variables				
Sex, n (%)				
Males	693 (74.5)	237 (25.5)	930 (100.0)	**0.006**
Females	907 (69.2)	404 (30.8)	1,311 (100.0)	
Cohort, n (%)				
Cohort 1	824 (72.1)	319 (27.9)	1,143 (100.0)	0.458
Cohort 2	776 (70.7)	322 (29.3)	1,098 (100.0)	
Age at baseline, mean (sd)	68.8 (1.4)	69.2 (1.5)	68.9 (1.5)	**<0.001**
Living alone, n (%)				
No	1,057 (75.1)	351 (24.9)	1,408 (100.0)	**<0.001**
Yes	541 (65.1)	290 (34.9)	831 (100.0)	
Marital status, n (%)				
Single	184 (66.4)	93 (33.6)	277 (100.0)	**<0.001**
Married	938 (74.8)	316 (25.2)	1,254 (100.0)	
Separated or divorced	295 (69.1)	132 (30.9)	427 (100.0)	
Widowed	178 (64.3)	99 (35.7)	277 (100.0)	
Number of children, n (%)				
0	320 (66.9)	158 (33.1)	478 (100.0)	0.061
1	247 (71.0)	101 (29.0)	348 (100.0)	
2	708 (73.6)	254 (26.4)	962 (100.0)	
3+	309 (72.9)	115 (27.1)	424 (100.0)	
Chronic conditions, n (%)				
0	445 (82.0)	98 (18.0)	543 (100.0)	**<0.001**
1	616 (74.4)	212 (25.6)	828 (100.0)	
2+	534 (61.9)	329 (38.1)	863 (100.0)	
Smoking history, n (%)				
Current	268 (69.1)	120 (30.9)	388 (100.0)	0.515
Former	637 (71.6)	253 (28.4)	890 (100.0)	
Never	685 (72.2)	264 (27.8)	949 (100.0)	
Problematic alcohol history, n (%)				
No	1,541 (72.1)	597 (27.9)	2,138 (100.0)	**<0.001**
Yes	49 (55.1)	40 (44.9)	89 (100.0)	
SES: Young adulthood				
Education, n (%)				
Basic compulsory	281 (65.2)	150 (34.8)	431 (100.0)	**0.003**
Apprenticeship	648 (71.7)	256 (28.3)	904 (100.0)	
Post-compulsory schooling	667 (74.1)	233 (25.9)	900 (100.0)	
SES: Adulthood				
Occupational class, n (%)				
Manager, self-employed, liberal prof., director	592 (72.6)	223 (27.4)	815 (100.0)	**0.002**
Skilled worker/employee, farmer	670 (74.4)	231 (25.6)	901 (100.0)	
Non-skilled worker/employee	236 (66.3)	120 (33.7)	356 (100.0)	
No professional activity	64 (60.4)	42 (39.6)	106 (100.0)	
SES: Early old age (subjective measures)				
Income, n (%)				
Clearly higher	51 (79.7)	13 (20.3)	64 (100.0)	**<0.001**
Rather higher	980 (74.6)	333 (25.4)	1,313 (100.0)	
Rather lower	434 (67.1)	213 (32.9)	647 (100.0)	
Clearly lower	73 (59.8)	49 (40.2)	122 (100.0)	
Wealth, n (%)				
Clearly higher	64 (79.0)	17 (21.0)	81 (100.0)	**<0.001**
Rather higher	857 (75.0)	285 (25.0)	1,142 (100.0)	
Rather lower	457 (67.9)	216 (32.1)	673 (100.0)	
Clearly lower	150 (64.1)	84 (35.9)	234 (100.0)	
Financial strain, n (%)				
No	1,421 (73.4)	514 (26.6)	1935 (100.0)	**<0.001**
Yes	176 (59.3)	121 (40.7)	297 (100.0)	
SES: Early old age (objective measure)				
Financial subsidies, n (%)				
No	1,360 (73.6)	488 (26.4)	1848 (100.0)	**<0.001**
Yes	218 (60.6)	142 (39.4)	360 (100.0)	

^a^ p-value from Pearson Chi-squared test or Student’s t-test.

Note: missing values: Education (6); Occupation (63), Income (95), Wealth (111), Financial subsidies (33), Financial strain (9), Living alone (2), Marital status (6), Number of children (29), chronic conditions (7), smoking history (14), problematic alcohol history (14).

As indicated in Table 2, all socioeconomic measures were associated with baseline frailty when they were analyzed in separate models and after adjustment for sex, age, cohort, living alone, marital status, and number of children. In a mutually adjusted model with similar adjustment, adjusted odds ratios (aOR) for baseline frailty were as follows: 1.44 (95% CI, 1.01–2.06) for basic compulsory education and 1.31 (95% CI, 1.01–1.70) for apprenticeship compared to post-compulsory schooling; 1.64 (95% CI, 1.01–2.65) for no professional activity compared to an occupational class corresponding to manager, self-employed, liberal profession, or director; 1.73 (95% CI, 1.30–2.30) for reporting financial strain; and 1.37 (95% CI, 1.04–1.80) for receiving financial subsidies.

**TABLE 2:** Logistic regression models: association between socioeconomic status measures and baseline frailty (Lausanne cohort 65+, Switzerland. 2004–2019).

Variables	Socioeconomic characteristics taken separately^a^ ^,^ ^b^		Mutually adjusted model for socioeconomic characteristics^b^ ^,^ ^c^	
	OR (95% CI)	p-value	OR (95% CI)	p-value
Socioeconomic status: Young adulthood				
Education				
Basic compulsory	1.49 (1.15–1.94)	**0.003**	1.44 (1.01–2.06)	**0.047**
Apprenticeship	1.17 (0.95–1.45)	0.146	1.31 (1.01–1.70)	0.039
Post-compulsory schooling	Ref.		Ref.	
Socioeconomic status: Adulthood				
Occupational class				
Manager, self-employed, liberal prof., director	Ref.		Ref.	
Skilled worker/employee, farmer	0.88 (0.71–1.11)	0.279	0.75 (0.58–0.98)	**0.034**
Non-skilled worker/employee	1.32 (0.99–1.75)	0.059	0.91 (0.62–1.33)	0.620
No professional activity	1.86 (1.19–2.92)	**0.007**	1.64 (1.01–2.65)	**0.044**
Socioeconomic status: Early old age (subjective measures)				
Income				
Clearly/rather higher	Ref.		d	
Clearly/rather lower	1.46 (1.20–1.79)	**<0.001**		
Wealth				
Clearly/rather higher	Ref.		d	
Clearly/rather lower	1.45 (1.19–1.77)	**<0.001**		
Financial strain				
No	Ref.		Ref.	
Yes	1.91 (1.47–2.49)	**<0.001**	1.73 (1.30–2.30)	**<0.001**
Socioeconomic status: Early old age (objective measure)				
Financial subsidies				
No	Ref.		Ref.	
Yes	1.72 (1.34–2.20)	**<0.001**	1.37 (1.04–1.80)	**0.024**

^a^ Six separate models: N = 2,200 (Education); N = 2,144 (Occupational class); N = 2,112 (Income); N = 2095 (Wealth); N = 2,197 (Financial strain); N = 2,174 (Financial subsidies).

^b^ Adjusted for sex, age, cohort, living alone, marital status, number of children.

^c^ One single model: N = 2,102.

^d^ To avoid overadjustment due to multiple subjective socioeconomic status, measures in early old age, income and wealth were not entered in the mutually adjusted model.

OR: odds ratio of (pre)frailty at baseline (reference: Non-frail).

**FIGURE 1:**
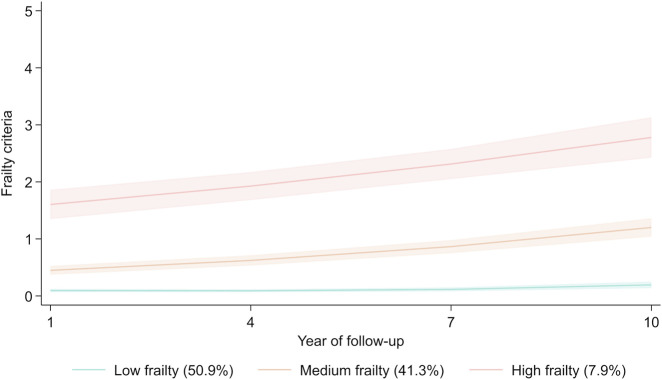
Frailty trajectories over 10 years (Lausanne cohort 65+, Switzerland. 2004–2019). Light shaded bands show 95% confidence intervals.

As regards frailty trajectories, a solution including three trajectories with one quadratic and two linear trajectories emerged as the best fitting and most parsimonious model (Figure 1). Trajectory 1 (low frailty, 51% of the sample) included individuals who remained predominantly non-frail over the 10-year follow-up period. Trajectory 2 (medium frailty, 41%) started with a low frailty score and progressed to pre-frailty with a score >1 at year 10. Trajectory 3 (high frailty, 8%) started at pre-frail level and showed a sharp increase towards frailty with a score close to 3 at year 10.

As indicated in Table 3, all measures of lower SES were associated with either medium or high frailty trajectories when they were analyzed in separate models and after adjustment for sex, age, cohort, living alone, marital status, number of children, and baseline frailty. In a mutually adjusted model with similar adjustment, increased odds for both medium and high trajectories were observed for reporting financial strain (aOR = 1.48 (95% CI, 1.07–2.05) and aOR = 2.28 (95% CI, 1.32–3.94), respectively) and for receiving financial subsidies (aOR = 1.69 (95% CI, 1.25–2.28) and aOR = 2.07 (95% CI, 1.22–3.51), respectively). By contrast, in mutually adjusted models, no associations were observed between frailty trajectories and education or occupational class.

**TABLE 3:** Multivariable analysis of the association between socioeconomic characteristics and frailty trajectories (Lausanne cohort 65+, Switzerland. 2004–2019).

Variables	Socioeconomic characteristics taken separately^a^ ^,^ ^b^				Mutually adjusted model for socioeconomic characteristics^b^ ^,^ ^c^			
	Medium trajectory		High trajectory		Medium trajectory		High trajectory	
	RRR (95% CI)	p-value	RRR (95% CI)	p-value	RRR (95% CI)	p-value	RRR (95% CI)	p-value
Socioeconomic status: Young adulthood								
Education								
Basic compulsory	1.16 (0.87–1.53)	0.305	2.21 (1.31–3.73)	**0.003**	0.81 (0.55–1.20)	0.290	1.58 (0.79–3.15)	0.198
Apprenticeship	1.18 (0.95–1.46)	0.143	1.79 (1.15–2.80)	**0.010**	1.16 (0.89–1.50)	0.278	1.46 (0.86–2.48)	0.163
Post-compulsory schooling	Ref.		Ref.		Ref.		Ref.	
Socioeconomic status: Adulthood								
Occupational class								
Manager, self-employed, liberal prof., director	Ref.		Ref.		Ref.		Ref.	
Skilled worker/employee, farmer	1.00 (0.80–1.26)	0.978	1.58 (1.00–2.52)	0.052	0.95 (0.72–1.24)	0.698	1.23 (0.71–2.11)	0.459
Non-skilled worker/employee	1.49 (1.10–2.02)	**0.011**	2.41 (1.35–4.29)	**0.003**	1.50 (0.99–2.26)	0.056	1.36 (0.64–2.88)	0.424
No professional activity	0.82 (0.48–1.39)	0.456	2.19 (0.94–5.07)	0.068	0.83 (0.47–1.45)	0.515	1.67 (0.67–4.15)	0.273
Socioeconomic status: Early old age (subjective measures)								
Income								
Clearly/rather higher	Ref.		Ref.		d		d	
Clearly/rather lower	1.36 (1.10–1.68)	**0.004**	1.46 (0.98–2.18)	0.061				
Wealth								
Clearly/rather higher	Ref.		Ref.		d		d	
Clearly/rather lower	1.53 (1.24–1.88)	**<0.001**	2.07 (1.38–3.09)	**<0.001**				
Financial strain								
No	Ref.		Ref.		Ref.		Ref.	
Yes	1.78 (1.32–2.41)	**<0.001**	2.63 (1.60–4.32)	**<0.001**	1.48 (1.07–2.05)	**0.017**	2.28 (1.32–3.94)	**0.003**
Socioeconomic status: Early old age (objective measure)								
Financial subsidies								
No	Ref.		Ref.		Ref.		Ref.	
Yes	1.89 (1.43–2.50)	**<0.001**	2.45 (1.51–3.96)	**<0.001**	1.69 (1.25–2.28)	**0.001**	2.07 (1.22–3.51)	**0.007**

^a^ Six separate models: N = 2,200 (Education); N = 2,144 (Occupational class); N = 2,112 (Income); N = 2095 (Wealth); N = 2,174 (Financial subsidies); N = 2,197 (Financial strain).

^b^ Adjusted for sex, age, cohort, living alone, marital status, number of children, baseline frailty.

^c^ One single model: N = 2,102.

^d^ To avoid overadjustment due to multiple subjective socioeconomic status, measures in early old age, income and wealth were not entered in the mutually adjusted model.

RRR: relative risk ratio (reference: low trajectory).

CI: confidence interval.

The sensitivity analysis, which included individuals lost to follow-up for reasons other than death or illness, involved a total of 2,562 individuals, compared to 2,241 in the main analysis. The shapes of frailty trajectories and the probabilities of belonging to a trajectory group closely mirrored those in the main analysis. Only 25 individuals (1.1%) were assigned to a different trajectory in the sensitivity analysis compared to the main analysis. Similarly, the associations between socioeconomic characteristics and frailty trajectories closely resembled those in the main analysis (refer to Supplementary Table S2). In the regression models that incorporated chronic conditions, smoking history, and problematic alcohol history as covariates, associations also remained essentially unchanged (refer to Supplementary Table S3).

## Discussion

### Main Findings

In this population of community-dwelling older adults, we found that measures of SES at different points of the life-course are all associated with worse frailty levels. A lower education, occupational class, and worse financial situation in older age are independently associated with higher frailty levels shortly after age 65. In addition, a worse financial situation in early old age is associated with both higher frailty levels as well as worse frailty trajectories after age 65. Findings suggest that SES inequalities in frailty are likely the results of influences across early, mid-life and later life stages, requiring interventions throughout the life-course. In addition, policies aimed at improving the financial situation of older people may help curb inequalities in frailty trajectories.

### Socioeconomic Inequalities in Baseline Frailty

The associations between socioeconomic characteristics and baseline frailty are consistent with previous studies indicating a particularly high prevalence of frailty in older people with lower education and income [23]. Three competing hypotheses summarize how these associations may change over the life course [24]: The “age-as-leveler” hypothesis posits that socioeconomic inequalities in health narrow in old age after a peak in mid-life or early old age. On the other hand, the “status maintenance” hypothesis suggests that inequalities that develop in early- or mid-life do not increase or decrease with age. Finally, the “cumulative advantage” hypothesis postulates that socioeconomic inequalities in health widen over the life course due to a progressive accumulation of disadvantages. In their literature review, Wang and Hulme compiled substantial evidence of socioeconomic inequalities in frailty [7], and claimed that “age-as-leveler” hypothesis was the prevailing pattern across most of the reviewed studies. While the present study was not specifically designed to formally identify an age pattern in socioeconomic inequalities, it is still interesting to note that SES measures across all life stages were independently associated with baseline frailty, suggesting that socioeconomic inequalities in frailty accumulate and are evident in individuals in their late sixties.

### Socioeconomic Inequalities in Frailty Trajectories

Previous studies aiming to differentiate multiple patterns of frailty trajectories have consistently identified three trajectories as the optimal model fit [10–12, 14, 25]. Our results similarly align with prior research indicating an association between unfavorable frailty trajectories and lower levels of education [10–12, 25] and occupational class [10]. Additionally, studies focusing on a single pattern of frailty change over time have shown that a linear or quadratic increase in frailty is linked with lower education and occupation [24, 26–28]. In line with our findings, Gardiner et al. [10] found that early-life SES measures were not independently associated with frailty trajectories when taking into account later-life SES measures. Other studies have also found that the pathways to the development of frailty begin early in life but are later mediated by socioeconomic factors [29].

In the present study, adjusting for the baseline level of frailty enabled us to isolate the factors specifically linked with the trajectory of frailty, and to determine whether ‘new’ inequalities emerge in older age, in addition to those generated by exposures earlier in life. We found that financial strain and the receipt of financial subsidies in older age were independently associated with the least favorable trajectories of frailty, whereas no associations were observed between educational level or occupational class and frailty trajectories in mutually adjusted models. One possible interpretation is that the associations for educational level and occupational class are mediated by financial measures of SES in later life. The financial situation in later life may affect frailty trajectories in multiple ways [30–32]. For instance, individuals experiencing financial strain may live in lower-quality housing that is less suited to supporting physical function maintenance, thereby creating an environment less favorable for healthy aging. Additionally, limited financial resources can restrict access to paid care and support services, potentially accelerating the decline in physical function. Other factors may include inadequate nutrition, limited access to physical activity opportunities, increased stress, reduced social engagement, and inability to afford preventive healthcare, indicating that financial stability is a key factor in mitigating frailty in older adults.

### Strengths and Limitations of the Study

A major strength of this study lies in its utilization of data from the Lc65+ study, which features a large and representative sample of community-dwelling older people. The Lc65+ study also provides multiple SES measures at various stages throughout the life-course. Frailty trajectories were assessed using a validated tool that combines both self-report and objective measures. However, some important limitations should be considered. First, merging pre-frail and frail individuals was necessary due to the low prevalence of frailty. This may be attributed to a lower occurrence of physical frailty in Switzerland compared to other European countries [33], as well as in studies employing Fried’s phenotype versus the cumulative deficit model [34]. Second, while the focus remained on physical frailty, it is worth noting that other conceptual definitions encompass additional dimensions such as psychological, social, and cognitive frailty. These dimensions may follow distinct trajectories, originating through different pathways. Third, our estimates lack a causal interpretation and solely focus on describing associations between SES and frailty. For example, it is possible that worse frailty trajectories could impact the financial situations of older households, potentially strengthening these associations. Future studies should explore the causal nature of the association between different SES measures and frailty trajectories.

### Implications for Public Health and Policy

Policies that break down class barriers rather than reinforce them—such as family-friendly social protection, inclusive education, and fair employment—can help reduce health inequalities and prevent their transmission across generations [35]. Research on income-support interventions, including cash transfer programs, income tax credits, and minimum wage policies, has shown modest but meaningful effects at the population level, with consistent long-term benefits [36]. However, further evaluations are needed to determine how these policies can be optimized for greater public health impact. Tackling socioeconomic inequalities early in life can help narrow disparities in frailty trajectories later on, ultimately supporting healthier aging for future generations.

While early-life interventions are crucial for reducing health inequalities, financial protection measures in older age also play a key role in promoting healthy aging. Policies aimed at securing adequate pension schemes, subsidized healthcare, and targeted social assistance can help mitigate the economic vulnerabilities that often intensify in later life [37]. Ensuring stable income and access to essential services can reduce stress, improve healthcare utilization, support social engagement, and promote overall wellbeing among older adults [38]. As populations continue to age, strengthening these financial protection mechanisms will be essential to fostering equity in health outcomes and preventing further socioeconomic-driven disparities in frailty.

### Conclusion

This study identified three distinct frailty trajectories in a Swiss population of community-dwelling older adults. We observed socioeconomic inequalities in frailty shortly after age 65, indicated by independent associations with educational level, occupational class, and both objective and subjective measures of financial situation in early old age. In addition, poorer financial situations in early old age were associated with worse frailty trajectories after age 65. Our findings suggest that addressing disparities in frailty requires interventions across the life-course. They also underscore the critical role of policies aimed at improving the financial wellbeing of older people in reducing SES inequalities in frailty trajectories during older age.

## References

[1] Fried LP Tangen CM Walston J Newman AB Hirsch C Gottdiener J Frailty in Older Adults: Evidence for a Phenotype. The journals Gerontol Ser A, Biol Sci Med Sci (2001) 56(3):M146–56. 10.1093/gerona/56.3.m146 11253156

[2] Vermeiren S Vella-Azzopardi R Beckwee D Habbig AK Scafoglieri A Jansen B Frailty and the Prediction of Negative Health Outcomes: A Meta-Analysis. J Am Med Directors Assoc (2016) 17(12):1163 e1–e17. 10.1016/j.jamda.2016.09.010 27886869

[3] Rockwood K Mitnitski A. Frailty Defined by Deficit Accumulation and Geriatric Medicine Defined by Frailty. Clin Geriatr Med (2011) 27(1):17–26. 10.1016/j.cger.2010.08.008 21093719

[4] Villacampa-Fernandez P Navarro-Pardo E Tarin JJ Cano A. Frailty and Multimorbidity: Two Related yet Different Concepts. Maturitas (2017) 95:31–5. 10.1016/j.maturitas.2016.10.008 27889050

[5] Roberts S Collins P Rattray M. Identifying and Managing Malnutrition, Frailty and Sarcopenia in the Community: A Narrative Review. Nutrients (2021) 13(7):2316. 10.3390/nu13072316 34371823 PMC8308465

[6] Lim WS Wong SF Leong I Choo P Pang WS. Forging a Frailty-Ready Healthcare System to Meet Population Ageing. Int J Environ Res Public Health (2017) 14(12):1448. 10.3390/ijerph14121448 29186782 PMC5750867

[7] Wang J Hulme C. Frailty and Socioeconomic Status: A Systematic Review. J Public Health Res (2021) 10(3):2036. 10.4081/jphr.2021.2036 33942603 PMC8477231

[8] Welstead M Jenkins ND Russ TC Luciano M Muniz-Terrera G. A Systematic Review of Frailty Trajectories: Their Shape and Influencing Factors. Gerontologist (2021) 61(8):e463–e475. 10.1093/geront/gnaa061 32485739 PMC8599181

[9] Kojima G Taniguchi Y Iliffe S Jivraj S Walters K. Transitions between Frailty States Among Community-Dwelling Older People: A Systematic Review and Meta-Analysis. Ageing Res Rev (2019) 50:81–8. 10.1016/j.arr.2019.01.010 30659942

[10] Gardiner PA Mishra GD Dobson AJ. The Effect of Socioeconomic Status across Adulthood on Trajectories of Frailty in Older Women. J Am Med Dir Assoc (2016) 17(4):372 e1–3. 10.1016/j.jamda.2015.12.090 26837597

[11] Jung Y Lyu J Kim G. Multi-Group Frailty Trajectories Among Older Koreans: Results From the Korean Longitudinal Study of Aging. Arch Gerontol Geriatr (2022) 98:104533. 10.1016/j.archger.2021.104533 34592680

[12] Hsu HC Chang WC. Trajectories of Frailty and Related Factors of the Older People in Taiwan. Exp Aging Res (2015) 41(1):104–14. 10.1080/0361073X.2015.978219 25494673

[13] Chamberlain AM St Sauver JL Jacobson DJ Manemann SM Fan C Roger VL Social and Behavioural Factors Associated with Frailty Trajectories in a Population-Based Cohort of Older Adults. BMJ Open (2016) 6(5):e011410. 10.1136/bmjopen-2016-011410 PMC4885446 27235302

[14] Peek MK Howrey BT Ternent RS Ray LA Ottenbacher KJ. Social Support, Stressors, and Frailty Among Older Mexican American Adults. J Gerontol B Psychol Sci Soc Sci (2012) 67(6):755–64. 10.1093/geronb/gbs081 23009957 PMC3478725

[15] Henchoz Y Blanco JM Fustinoni S Nanchen D Bula C Seematter-Bagnoud L Cohort Profile: The Lausanne Cohort 65+ (Lc65+). Int J Epidemiol (2022) 51(4):e156–e166. 10.1093/ije/dyab245 34849932 PMC9365621

[16] Santos-Eggimann B Karmaniola A Seematter-Bagnoud L Spagnoli J Bula C Cornuz J The Lausanne Cohort Lc65+: A Population-Based Prospective Study of the Manifestations, Determinants and Outcomes of Frailty. BMC Geriatr (2008) 8:20. 10.1186/1471-2318-8-20 18706113 PMC2532683

[17] Dent E Kowal P Hoogendijk EO. Frailty Measurement in Research and Clinical Practice: A Review. Eur J Intern Med (2016) 31:3–10. 10.1016/j.ejim.2016.03.007 27039014

[18] Bouillon K Kivimaki M Hamer M Sabia S Fransson EI Singh-Manoux A Measures of Frailty in Population-Based Studies: An Overview. BMC Geriatr (2013) 13:64. 10.1186/1471-2318-13-64 23786540 PMC3710231

[19] UNESCO. International Standard Classification of Education. Paris, France: UNESCO Institute for Statistics (2011). Available online at: http://www.uis.unesco.org/Education/Pages/international-standard-classification-of-education.aspx (Accessed 26 October, 2016).

[20] Fustinoni S Santos-Eggimann B Henchoz Y. Trajectories of Phenotypical Frailty over a Decade in Young-Old Community-Dwelling Adults: Results from the Lc65+ Study. J Epidemiol Community Health (2022) 76(3):216–22. 10.1136/jech-2021-216633 34433618

[21] Haviland AM Jones BL Nagin DS. Group-based Trajectory Modeling Extended to Account for Nonrandom Participant Attrition. Sociol Method Res (2011) 40(2):367–90. 10.1177/0049124111400041

[22] Zimmer Z Martin LG Nagin DS Jones BL. Modeling Disability Trajectories and Mortality of the Oldest-Old in China. Demography (2012) 49(1):291–314. 10.1007/s13524-011-0075-7 22246796

[23] Majid Z Welch C Davies J Jackson T. Global Frailty: The Role of Ethnicity, Migration and Socioeconomic Factors. Maturitas (2020) 139:33–41. 10.1016/j.maturitas.2020.05.010 32747038 PMC8054560

[24] Stolz E Mayerl H Waxenegger A Rasky E Freidl W. Impact of Socioeconomic Position on Frailty Trajectories in 10 European Countries: Evidence from the Survey of Health, Ageing and Retirement in Europe (2004-2013). J Epidemiol Community Health (2017) 71(1):73–80. 10.1136/jech-2016-207712 27422980

[25] Howrey BT Al SS Middleton JA Ottenbacher KJ. Trajectories of Frailty and Cognitive Decline Among Older Mexican Americans. J Gerontol A Biol Sci Med Sci (2020) 75(8):1551–7. 10.1093/gerona/glz295 32012218 PMC7357582

[26] Hoogendijk EO Heymans MW Deeg DJH Huisman M. Socioeconomic Inequalities in Frailty Among Older Adults: Results from a 10-Year Longitudinal Study in the Netherlands. Gerontology (2018) 64(2):157–64. 10.1159/000481943 29055946 PMC5841137

[27] Dugravot A Fayosse A Dumurgier J Bouillon K Rayana TB Schnitzler A Social Inequalities in Multimorbidity, Frailty, Disability, and Transitions to Mortality: A 24-year Follow-Up of the Whitehall II Cohort Study. Lancet Public Health (2020) 5(1):e42–e50. 10.1016/S2468-2667(19)30226-9 31837974 PMC7098476

[28] Soler-Vila H Garcia-Esquinas E Leon-Munoz LM Lopez-Garcia E Banegas JR Rodriguez-Artalejo F. Contribution of Health Behaviours and Clinical Factors to Socioeconomic Differences in Frailty Among Older Adults. J Epidemiol Community Health (2016) 70(4):354–60. 10.1136/jech-2015-206406 26567320

[29] Van der Linden BWA Cheval B Sieber S Orsholits D Guessous I Stringhini S Life Course Socioeconomic Conditions and Frailty at Older Ages. J Gerontol B Psychol Sci Soc Sci (2020) 75(6):1348–57. 10.1093/geronb/gbz018 30753721 PMC7265806

[30] Saravanakumar P Balachandran A Muhammad T Drishti D Srivastava S. Wealth Disparity and Frailty Among Community-Dwelling Older Adults in India. BMC Public Health (2022) 22(1):2123. 10.1186/s12889-022-14434-9 36401189 PMC9675126

[31] Hubbard RE Goodwin VA Llewellyn DJ Warmoth K Lang IA. Frailty, Financial Resources and Subjective Well-Being in Later Life. Arch Gerontol Geriatr (2014) 58(3):364–9. 10.1016/j.archger.2013.12.008 24512820

[32] Lang IA Hubbard RE Andrew MK Llewellyn DJ Melzer D Rockwood K. Neighborhood Deprivation, Individual Socioeconomic Status, and Frailty in Older Adults. J Am Geriatr Soc (2009) 57(10):1776–80. 10.1111/j.1532-5415.2009.02480.x 19754500

[33] Santos-Eggimann B Cuenoud P Spagnoli J Junod J. Prevalence of Frailty in Middle-Aged and Older Community-Dwelling Europeans Living in 10 Countries. J Gerontol A Biol Sci Med Sci (2009) 64(6):675–81. 10.1093/gerona/glp012 19276189 PMC2800805

[34] Collard RM Boter H Schoevers RA Oude Voshaar RC. Prevalence of Frailty in Community-Dwelling Older Persons: A Systematic Review. J Am Geriatr Soc (2012) 60(8):1487–92. 10.1111/j.1532-5415.2012.04054.x 22881367

[35] Houweling TAJ Grunberger I. Intergenerational Transmission of Health Inequalities: Towards a Life Course Approach to Socioeconomic Inequalities in Health - a Review. J Epidemiol Community Health (2024) 78(10):641–9. 10.1136/jech-2022-220162 38955463 PMC11420752

[36] Boccia D Maritano S Pizzi C Richiardi MG Lioret S Richiardi L. The Impact of Income-Support Interventions on Life Course Risk Factors and Health Outcomes during Childhood: A Systematic Review in High Income Countries. BMC Public Health (2023) 23(1):744. 10.1186/s12889-023-15595-x 37087420 PMC10121417

[37] Eozenou PH Neelsen S Smitz MF. Financial Protection in Health Among the Elderly - A Global Stocktake. Health Syst Reform (2021) 7(2):e1911067. 10.1080/23288604.2021.1911067 34402386

[38] McMaughan DJ Oloruntoba O Smith ML. Socioeconomic Status and Access to Healthcare: Interrelated Drivers for Healthy Aging. Front Public Health (2020) 8:231. 10.3389/fpubh.2020.00231 32626678 PMC7314918

