# Supplementary material for: Socioeconomic Status Across the Life-Course and Frailty in Older Age: Evidence From Switzerland
Source: Int J Public Health. 2025 Jun 9;70:1608102. doi: 10.3389/ijph.2025.1608102 (PMC12183461; doi:10.3389/ijph.2025.1608102)
Supplement: Supplementary file 1 [file DataSheet1.pdf]

Supplementary material

Supplementary Figure S1. Selection procedure of study participants (Lausanne cohort 65+, Switzerland. 2004-2019).

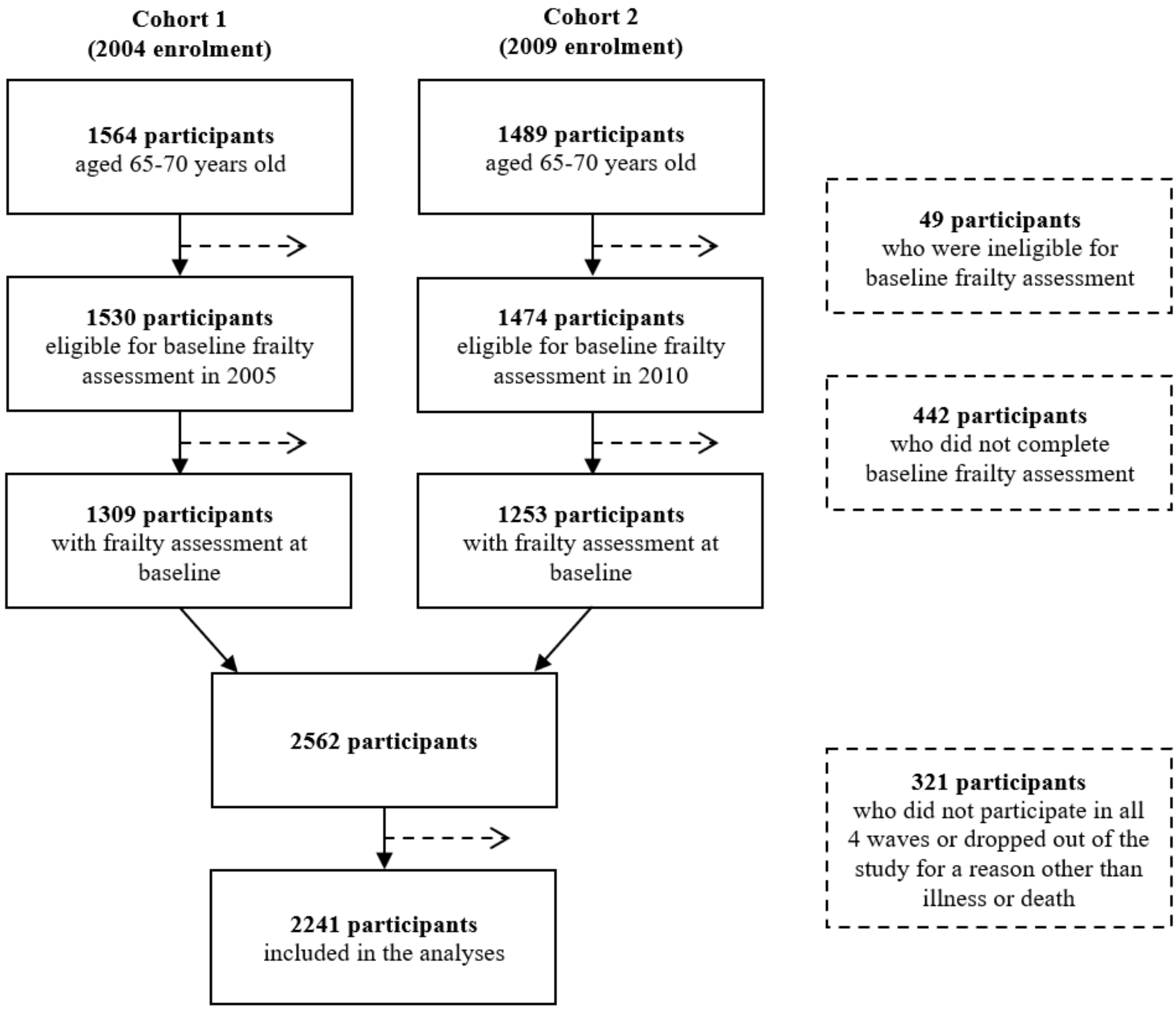

Supplementary Table S1. Comparison between included and excluded participants (Lausanne cohort 65+, Switzerland. 2004-2019).

	Included<br>n=2241	Excluded<br>n=812	Total<br>N=3053	p-value[a]
Sex, n (%)				
Males	930 (41.5)	330 (40.6)	1260 (41.3)	.670
Females	1311 (58.5)	482 (59.4)	1793 (58.7)	
Cohort, n (%)				
Cohort 1	1143 (51.0)	421 (51.8)	1564 (51.2)	.680
Cohort 2	1098 (49.0)	391 (48.2)	1489 (48.8)	
Age at baseline, mean (sd)	68.9 (1.4)	69.1 (1.4)	68.9 (1.4)	**.003**
Subjective health, n (%)				
Very good, good	1541 (69.0)	438 (54.1)	1979 (65.0)	**<.001**
Average, poor, very poor	693 (31.0)	371 (45.9)	1064 (35.0)	

[a]p-value from Pearson's Chi-square test

Note: missing values: subjective health (10)

Supplementary Table S2. Sensitivity analysis: Multivariable analysis of the association between socioeconomic characteristics and frailty trajectories (ref. = Low trajectory), including individuals lost to follow-up for reasons other than death or illness (Lausanne cohort 65+, Switzerland. 2004-2019).

	Socioeconomic characteristics taken separately [ac]				Mutually adjusted model for socioeconomic characteristics[bc]			
	Medium trajectory		High trajectory		Medium trajectory		High trajectory	
	RRR (95% CI)	p-value	RRR (95% CI)	p-value	RRR (95% CI)	p-value	RRR (95% CI)	p-value
**SES: Young adulthood**								
Education								
Basic compulsory	1.20 (0.93 – 1.56)	.160	1.80 (1.10 – 2.94)	**.019**	0.85 (0.59 – 1.21)	.362	1.19 (0.62 – 2.28)	.595
Apprenticeship	1.16 (0.94 – 1.44)	.160	1.66 (1.08 – 2.57)	**.022**	1.06 (0.82 – 1.36)	.668	1.31 (0.78 – 2.18)	.305
Post-compulsory schooling	Ref.		Ref.		Ref.		Ref.	
**SES: Adulthood**								
Occupational class								
Manager, self-employed, liberal prof., director	Ref.		Ref.		Ref.		Ref.	
Skilled worker/employee, farmer	1.12 (0.90 – 1.39)	.316	1.71 (1.09 – 2.70)	**.020**	1.09 (0.85 – 1.42)	.492	1.41 (0.83 – 2.38)	.203
Non-skilled worker/employee	1.58 (1.19 – 2.10)	**.002**	2.15 (1.25 – 3.73)	**.006**	1.57 (1.06 – 2.30)	**.023**	1.49 (0.73 – 3.01)	.270
No professional activity	0.85 (0.51 – 1.44)	.555	2.35 (1.06 – 5.22)	**.036**	0.88 (0.51 – 1.52)	.650	1.82 (0.77 – 4.34)	.175
**SES: Early old age (subjective measures)**								
Income								
Clearly/rather higher	Ref.		Ref.		d		d	
Clearly/rather lower	1.29 (1.05 – 1.57)	**.014**	1.34 (0.92 – 1.97)	.131				
Wealth								
Clearly/rather higher	Ref.		Ref.		d		d	
Clearly/rather lower	1.38 (1.14 – 1.68)	**.001**	2.05 (1.39 – 3.03)	**<.001**				
Financial strain								
No	Ref.		Ref.		Ref.		Ref.	
Yes	1.84 (1.38 – 2.44)	**<.001**	2.71 (1.69 – 4.34)	**<.001**	1.50 (1.11 – 2.03)	**.008**	2.35 (1.41 – 3.93)	**.001**
**SES: Early old age (objective measure)**								
Financial subsidies								
No	Ref.		Ref.		Ref.		Ref.	
Yes	1.87 (1.44 – 2.41)	**<.001**	2.23 (1.42 – 3.49)	**<.001**	1.69 (1.28 – 2.23)	**<.001**	1.95 (1.19 – 3.19)	**.008**

[a] Six separate models: N=2508 (Education); N=2440 (Occupational class); N=2398 (Income); N=2378 (Wealth); N=2503 (Financial Strain); N=2480 (Financial subsidies).
[b] One single model: N=2392
[c] adjusted for sex, age, cohort, living alone, marital status, number of children, baseline frailty.
[d] To avoid overadjustment due to multiple subjective SES measures in early old age, income and wealth were not entered in the mutually adjusted model.
SES: socioeconomic status
RRR: relative risk ratio (reference: low trajectory)
CI: confidence interval

Supplementary Table S3. Sensitivity analysis: Multivariable analysis of the association between socioeconomic characteristics and frailty trajectories (ref. = Low trajectory), adjusted for chronic conditions, smoking history, and problematic alcohol history (Lausanne cohort 65+, Switzerland. 2004-2019).

	Socioeconomic characteristics taken separately [ac]				Mutually adjusted model for socioeconomic characteristics [bc]			
	Medium trajectory		High trajectory		Medium trajectory		High trajectory	
	RRR (95% CI)	p-value	RRR (95% CI)	p-value	RRR (95% CI)	p-value	RRR (95% CI)	p-value
**SES: Young adulthood**								
Education								
Basic compulsory	1.12 (0.84 – 1.50)	.438	2.05 (1.19 – 3.55)	**.010**	0.79 (0.53 – 1.18)	.252	1.52 (0.74 – 3.14)	.258
Apprenticeship	1.16 (0.93 – 1.45)	.200	1.63 (1.02 – 2.59)	**.039**	1.12 (0.86 – 1.47)	.394	1.30 (0.75 – 2.26)	.356
Post-compulsory schooling	Ref.		Ref.		Ref.		Ref.	
**SES: Adulthood**								
Occupational class								
Manager, self-employed, liberal prof., director	Ref.		Ref.		Ref.		Ref.	
Skilled worker/employee, farmer	1.00 (0.79 – 1.26)	.995	1.51 (0.93 – 2.45)	.095	0.96 (0.73 – 1.27)	.779	1.25 (0.71 – 2.20)	.444
Non-skilled worker/employee	1.49 (1.08 – 2.04)	**.014**	2.34 (1.27 – 4.30)	**.006**	1.55 (1.02 – 2.37)	**.041**	1.43 (0.65 – 3.17)	.373
No professional activity	0.75 (0.44 – 1.30)	.306	2.25 (0.94 – 5.38)	.067	0.79 (0.44 – 1.39)	.409	1.83 (0.70 – 4.73)	.216
**SES: Early old age (subjective measures)**								
Income								
Clearly/rather higher	Ref.		Ref.		d		d	
Clearly/rather lower	1.26 (1.02 – 1.57)	**.036**	1.25 (0.82 – 1.89)	.304				
Wealth								
Clearly/rather higher	Ref.		Ref.		d		d	
Clearly/rather lower	1.43 (1.15 – 1.76)	**.001**	1.60 (1.05 – 2.45)	**.028**				
Financial strain								
No	Ref.		Ref.		Ref.		Ref.	
Yes	1.64 (1.20 – 2.25)	**.002**	2.43 (1.45 – 4.08)	**.001**	1.40 (1.01 – 1.96)	**.046**	2.29 (1.30 – 4.02)	**.004**
**SES: Early old age (objective measure)**								
Financial subsidies								
No	Ref.		Ref.		Ref.		Ref.	
Yes	1.64 (1.23 – 2.20)	**.001**	2.13 (1.29 – 3.51)	**.003**	1.48 (1.09 – 2.01)	**.013**	1.80 (1.04 – 3.10)	**.035**

[a] Six separate models: N=2174 (Education); N=2120 (Occupational class); N=2088 (Income); N=2071 (Wealth); N=2170 (Financial subsidies); N=2152 (Financial strain).
[b] One single model: N=2084
[c] Adjusted for sex, age, cohort, living alone, marital status, number of children, chronic conditions, smoking history, problematic alcohol history, baseline frailty.
[d] To avoid overadjustment due to multiple subjective SES measures in early old age, income and wealth were not entered in the mutually adjusted model.
SES: socioeconomic status
RRR: relative risk ratio (reference: low trajectory)
CI: confidence interval
